# Roles of SlETR7, a newly discovered ethylene receptor, in tomato plant and fruit development

**DOI:** 10.1038/s41438-020-0239-y

**Published:** 2020-02-01

**Authors:** Yi Chen, Guojian Hu, Celeste Rodriguez, Meiying Liu, Brad M. Binder, Christian Chervin

**Affiliations:** 1 College of Food and Pharmaceutical Sciences, Ningbo University, Ningbo, China; 2 Genomics and Biotechnology of Fruits, University of Toulouse, INRA, ENSAT, 31326 Castanet-Tolosan, France; 3 Department of Biochemistry & Cellular and Molecular Biology, University of Tennessee, Knoxville, TN USA; 4 Weifang University, Weifang, 261041 Shandong China

**Keywords:** Plant signalling, Plant hormones

## Abstract

Ethylene regulates many aspects of plant growth and development. It is perceived by a family of ethylene receptors (ETRs) that have been well described. However, a full understanding of ETR function is complicated by functional redundancy between the receptor isoforms. Here, we characterize a new ETR, SlETR7, that was revealed by tomato genome sequencing. *SlETR7* expression in tomato fruit pericarp increases when the fruit ripens and its expression is synchronized with the expression of *SlETR1*, *SlETR2*, and *SlETR5* which occurs later in the ripening phase than the increase observed for *SlETR3*, *SlETR4*, and *SlETR6*. We uncovered an error in the *SlETR7* sequence as documented in the ITAG 3 versions of the tomato genome which has now been corrected in ITAG 4, and we showed that it belongs to sub-family II. We also showed that SlETR7 specifically binds ethylene. Overexpression (OE) of *SlETR7* resulted in earlier flowering, shorter plants, and smaller fruit than wild type. Knock-out (KO) mutants of *SlETR7* produced more ethylene at breaker (Br) and Br + 2 days stages compared to wild type (WT), but there were no other obvious changes in the plant and fruit in these mutant lines. We observed that expression of the other *SlETRs* is upregulated in fruit of *SlETR7* KO mutants, which may explain the absence of obvious ripening phenotypes. Globally, these results show that SlETR7 is a functional ethylene receptor. More work is needed to better understand its specific roles related to the six other tomato ETRs.

## Introduction

Fruits are important crops for world food security. The control of fruit ripening has attracted the attention of many scientists because poor fruit preservation contributes to the yearly one billion tons of food losses^1^. Ethylene is a key player in fruit development^2,3^ and this phytohormone has important roles from fruit set to ripening. Ethylene also regulates many other aspects of plant development such as seed germination, growth, and flower development, as well as plant responses to biotic and abiotic stresses^4^. In plants, ethylene is perceived by a family of ethylene receptor proteins (ETRs) localized in the endoplasmic reticulum membrane^5^. Over the past 20+ years, many ethylene receptor genes have been identified in different plant species^5^, as well as in a cyanobacterium^6^.

In tomato, which is studied as a model fleshy fruit, seven ethylene receptors have been reported. Indeed, SlETR1 through SlETR5 directly bind ethylene^7^, but SlETR6 and SlETR7 have not been tested. Gain-of-function mutations in either *SlETR1* or *SlETR3* give rise to tomato plants that are less sensitive to ethylene and these mutations also delay fruit ripening^8,9^. The downregulation of either *SlETR4* or *SlETR6* expression results in early fruit ripening^10,11^. Together, these data indicate that SlETRs have important roles in fruit ripening.

ETR functional redundancy and sub-functionalization offer to the plants a wide array of responses^12^. However, since plants contain multiple ethylene receptor isoforms^13^, it is often difficult to determine the function of a single receptor because of this functional redundancy. For example, in tomato, SlETR3 (also known as Never-Ripe or NR) and SlETR4 have been shown to compensate functionally for the other, where knock-down of *SlETR3* results in increased expression of *SlETR4*^14^. Adding to this problem is the fact that *SlETR* expression is variable during fruit ripening^11,13,15,16^. However, the *SlETR* expression kinetics were analyzed over a limited number of stages of fruit ripening from breaker to red fruit. Thus, having, a finer resolution of the kinetics of *SlETR* expression (e.g., day-to-day changes), which has never been recorded, may bring more information about the fine tuning of *SlETRs* during ripening and their role in this process.

Recent studies have reported a new ethylene receptor (*SlETR7*) in tomato^13,15^. In order to gain a more complete understanding about the ethylene receptors during tomato fruit ripening, and to position *SlETR7* expression in this refined pattern of regulation, we examined the changes in *ETR* expression during fruit ripening with higher time resolution from immature green to breaker + 7 days. This included day-by-day analysis from breaker stage onward. We also looked at the expression levels of the *ETRs* in non-fruit tissues. Since no prior study has determined whether or not SlETR7 is a functional ethylene receptor, we first tested its ability to bind ethylene. We then generated knock-out (KO) and overexpression (OE) lines for *SlETR7*. Together, these studies reveal that SlETR7 is a functional ethylene receptor.

## Methods

### Plant material, growth conditions

Tomato (*Solanum lycopersicum, cv*. Micro-tom) seeds were sterilized with 5% NaClO for 10 min and washed with sterilized water for 3–4 times. Then seeds were germinated in 1/2 strength Murashige and Skoog (MS) medium and 10-day-old seedlings were transferred to soil, grown in a greenhouse on a 16 h:8 h light:dark cycle where temperature during the day was 22 °C and at night was 18 °C. The light intensity during the day was 250 µmol m^−2^ s^−1^ and the relative humidity was maintained at 80%. To study fruit development and ripening, the anthesis flowers were tagged and fruits were analyzed at different stages: IMG (Immature green), MG (Mature green), Br (breaker), Br2 (Breaker + 2 days), Br5 (Breaker + 5 days), Br8 (Breaker + 8 days). The stages of fruit development were determined by the number of days following the flower anthesis for the immature stages: IMG was 25 days after anthesis and MG was 38 days after anthesis. Then from breaker stage, when the first yellow patches appear on green fruit, the stages were described by the number of days following the breaker stage. All sampling was performed in the morning, 2 hours after lights were switched on. For RNA extraction from different parts of the plant, the roots, stem, and fourth leaf from the top of 30-day old seedlings were sampled. Prior to extraction the roots were cut and rinsed three times with sterile water.

### Transgenic plant construction

SlETR7 KO mutant and Over Expressor (OE) plants were generated in this study. Details and sequences are given in Supplementary Fig. S1. KO mutants were generated according to Brooks et al.^17^. Two sgRNAs were designed with the CRISPR-P tool (http://crispr.hzau.edu.cn/CRISPR/). These sgRNAs (Supplementary Fig. S1A) were targeted at 36 bp and 155 bp after the translation start site, located in the first transmembrane domain of the SlETR7 protein. The plasmid was assembled by the Golden Gate strategy as described by Brooks et al.^17^. To obtain SlETR7 overexpressor (OE), the full-length of SlETR7 was amplified from Micro-tom leaf cDNA with the forward 5′-CATGCCATGGATGGCTACTGATAGTGAGTTCTCCAAT-3′ and the reverse 5′-CGAGCTCTTAAAAGCCTTCACCAGCTCT-3′ primers. The PCR product was digested with NcoI-SacI restriction enzyme and inserted into pGreen vector. The SlETR7 is driven by 2 × 35S promoter in pGreen vector. The construct was confirmed by sequencing.

Two plasmids were transformed into tomato mediated by *Agrobacterium tumefaciens* (C58). Both KO and OE plant transformation were selected with the antibiotic kanamycin (100 mg L^−1^). Two independent KO lines (*KO-L1* and *KO-L2*) were chosen from 20 positive T0 plants, and we also chose two independent OE lines (*OE-L1* and *OE-L2*) using a similar selection scheme with the OE construct.

### Bioinformatics

The phylogenetic tree was constructed in MEGA 6.06. Multiple alignments of the full-length ETR protein sequences from Arabidopsis, tomato and rice were performed with MUSCLE. The MUSCLE alignments was used for constructing the phylogenetic tree using the maximum likelihood method. Bootstrap analysis was performed using 1000 replicates. Regarding the *ETR* gene expression profiles in tomato, a heat map was generated with Clustvis^18^ using the mean of qPCR data for each *ETR* at each development stage. Statistical tests were performed with the R software (https://www.r-project.org/).

### Heterologous expression of SlETR7 and ethylene binding assays

To identify SlETR7, the full-length of *SlETR7* was cloned and sequenced (Supplementary Fig. S2). The number of transmembrane domains of SlETR7 was predicted by TMpred (https://embnet.vital-it.ch/software/TMPRED_form.html) and the sequence of *SlETR7* encoding the first 130 amino acids (1–390 bp) was condon-optimized by Integrated DNA Technologies (https://eu.idtdna.com/CodonOpt) and then synthesized for expression in *Pichia pastoris*. This sequence was fused to the coding sequence of glutathione S-transferase and introduced into the pPICZ A vector with EcoRI, KpnI and NotI restriction enzymes. We designate this construct (SlETR7[1–130]GST). Additionally, GST alone was introduced into pPICZ A as a negative control. Ethylene binding assays were performed on whole cells as previously described^19^ to determine total ethylene binding versus non-specific ethylene binding levels.

### Effects of ethylene on dark-grown seedlings

To measure the effects of ethylene on seedling growth, seeds of WT, KO-L1, KO-L2, OE-L1, and OE-L2 were surface sterilized with 5% (w/v) sodium hypochlorite for 10 min and washed 3–4 times with sterilized water. To ensure the seeds germinated at the same time, seeds were gently shaken (50 rpm) in distilled water overnight at 25 °C. At the stage of radicle protrusion, seeds were transferred to agar plates containing 1/2 strength MS medium, pH 5.9, 0.8% (w/v) agar, with no added sugar. Seedlings were then grown in a dark room at 28 °C vertically for 6 days in air or 0.1, 1, 10 or 100 ppm ethylene. Images were then acquired and hypocotyl and root lengths measured with ImageJ (version 1.51j8). To test whether *SlETR7* affected hook angle in tomato seedlings, 3-day-old seedlings grown in the dark were treated with 1 ppm ethylene. Hook angle was recorded over time at 0, 3, 6, and 18 h after treatment.

### Fruit development indices

Fruit weight and width were measured in Br7 fruit with at least eight fruits per line analyzed. Firmness was assessed with Harpenden calipers (British Indicators Ltd.) at IMG, MG, Br, Br2, Br5, and Br8 stages, as described previously^20^. Fruit color changes were measured with a chromameter (CR400, Konica Minolta) in IMG, MG, Br, Br2, and Br5. For ethylene production measurements, at least five fruits per stage were harvested. Fruits were left on the bench for 1 h to avoid ethylene induced by picking stress. Then each fruit was incubated in a 125 ml glass bottle for 1 h at which time a 1 ml sample was taken and analyzed by gas chromatography as described previously^21^.

### RNA purification and qPCR

For checking *SlETR* gene expression patterns, samples were taken from roots, stems, leaves, flowers and fruits at different stages of development. All samples were frozen with liquid nitrogen immediately after harvest and stored at −80 °C. Samples were the homogenized to a powder with a ball grinder. Fifty milligrams of sample was used for extracting RNA with Promega RNA kit. The total RNA sample was treated with DNAseI (Ambion) to remove DNA. One-microgram RNA was used for reverse transcription using the Promega RT protocol. qPCR was performed as described previously^22^. All the primers used for qPCR are listed in Supplementary Fig. S4.

## Results

### Expression of the *ETRs* in fruits during development and vegetative tissues

**Fig. 1:**
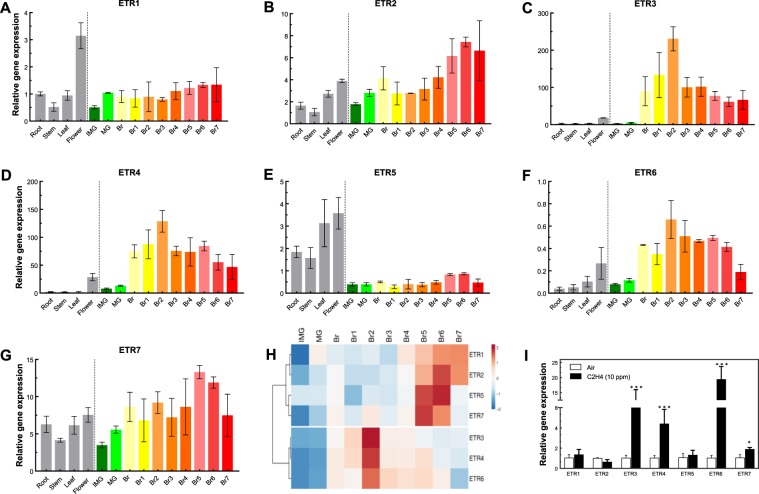
Gene expression profile of ETRs in tomato. **a**–**g** Total RNA was isolated from different tissues (root, stem, leaf, and flower) and fruit pericarp + skin at different development stages (IMG, immature green; MG, mature green; Br, breaker; Br1, breaker + 1 day to Br7, breaker + 7 days). The RNA expression of all *SlETRs* were calculated relative to *ETR1* levels in the root by using *Actin* and *EF1α* as reference genes. Data show the mean ± SD, *n* = 3; **h** heat map of clustering analysis of *SlETRs* expression during fruit development and ripening. **i** qPCR analysis of *SlETR* expression in response to 10 ppm exogenous ethylene in 2-week old seedlings. Data represent the mean ± SD, *n* = 3. Statistical analyses were performed using *t*-test comparing the air treatment with ethylene treatment, *P < 0.05, ***P < 0.001

The expression of the seven *SlETRs* was analyzed in root, stem, leaf, flower and developing fruit. In this particular series of experiments, the fruit samples were harvested day by day from the breaker stage to breaker + 7 days. All the data were normalized to *SlETR1* abundance in root. We observed *ETR* expression in all tissues tested. In tissues other than fruit, the highest expression of *SlETRs* occurred in flowers (Fig. 1a–g).

During fruit ripening *SlETR3* and *SlETR4* have higher expression levels than the other five *SlETRs* (Fig. 1a–g). SlETR7 is the third highest expressed ethylene receptor during fruit ripening followed by *SlETR2* and *SlETR1*. The *SlETR5* and *SlETR6* are the least expressed in fruit. Based on the gene expression patterns during fruit development and ripening, the receptors can be classified into two groups. One group (*SlETR3*, *SlETR4*, *SlETR6*) has a peak in gene expression at Br + 2 of fruit ripening. By contrast, the second group (*SlETR1*, *SlETR2*, *SlETR5*, *SlETR7*) has a peak in gene expression at a later stage of fruit ripening that occurred around Br + 5 or Br + 6. These differences in the timing of peak gene expression are highlighted in the co-expression analysis heatmap shown in Fig. 1h.

We were also curious to know whether or not the expression of the *SlETRs* was affected by ethylene. To test this, 2-week-old seedlings were treated with 10 ppm ethylene for 3 hours and qPCR carried out. *SlETR3*, *SlETR4* and *SlETR6* are ethylene responsive (Fig. 1i) as previously shown in immature green fruit^11^. Additionally, *SlETR7* expression is increased by ethylene treatment. However, of the four ethylene-induced receptors, it shows the smallest response (Fig. 1i). It is interesting to note that the three receptor isoforms that have a peak in gene expression earlier in fruit ripening are also the three receptor isoforms that are the most induced by ethylene with the other four either not induced or minimally induced by ethylene. These results suggest that the two groups of *SlETRs* are differentially regulated and may have different roles in fruit ripening.

### Identification of SlETR7 in tomato and ethylene binding activity

We have previously identified SlETR7 as a putative ethylene receptor^13,15^ and the above results suggests it might be involved in tomato fruit ripening. Because of this, we next focused on SlETR7 (Solyc05g055070).

**Fig. 2:**
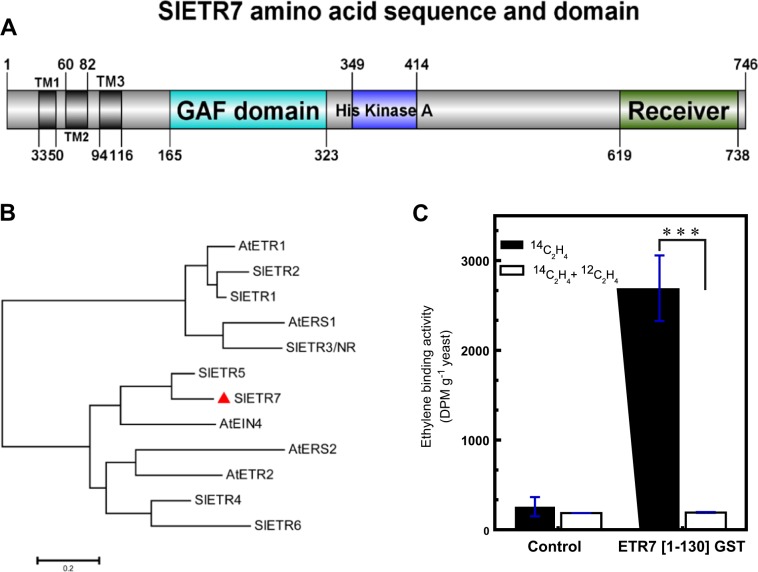
Tomato contains a seventh ethylene receptor. **a** Schematic structure of SlETR7 protein. Three conserved transmembrane domains are predicted at N-terminus followed by GAF, kinase, and receiver domains using TMpred and SMART tools. **b** Phylogenetic tree analysis of ETR proteins in Arabidopsis, tomato, and rice. The scale bar presents the substitution per amino acid based on bootstrap method (*B* = 1000 replications). The accessions of ETR proteins used in this analysis are: AtETR1 (NP_176808), AtERS1 (NP_181626), AtERS2 (NP_001323287), AtEIN4 (NP_187108), AtETR2 (XP_002883407), SlETR1 (NP_001234149), SlETR2 (NP_001234153), SlETR3 (NP_001233894), SlETR4 (NP_001234205), SlETR5 (NP_001234212), SlETR6 (NP_001234150). The amino acid sequence was translated from the coding region (Supplementary S2C). **c** Ethylene binding assays were conducted on *P. pastoris* cells expressing either GST alone (GST) as a negative control or expressing the first 130 amino acids of SlETR7 fused to GST (ETR7[1–130]GST). Data represent the mean ± SD, *n* = 3. The “***” indicates a significant difference using a *t*-test (*P* < 0.001) between the binding levels with ^14^C-ethylene alone versus ^14^C-ethylene plus excess of ^12^C-ethylene

Differences have been previously noted in this gene, based on the different versions of the genome sequence. This initially resulted in a prediction of four transmembrane domains by Liu et al.^15^ when using ITAG 3.0. Before this, using the ITAG 2.3 version of the *SlETR7* sequence, three transmembrane domains were predicted to occur in SlETR7 using the TMpred tool. To further examine this, the full-length of *SlETR7* was amplified from cDNA and cloned into the pGEM-T vector and then was sequenced. This sequencing (Supplementary Fig. S2B) revealed that there are an additional 54 nucleotides in this gene in the ITAG 3 cDNA versions, compared to the sequence we cloned (Supplementary Fig. S2A). The error in *SlETR7* cDNA has now been corrected in the ITAG 4 version. From the predicted amino acid sequence, we determined the putative domain structure of SlETR7. As shown in Fig. 2a, the SlETR7 protein is predicted to contain three transmembrane domains (33 aa − 116 aa), that were confirmed with the TMpred tool, followed by a GAF domain (165 aa − 323 aa), His kinase A domain (349 aa − 414 aa), and receiver domain (619 aa − 748 aa). Thus, this protein has a predicted domain organization similar to other ethylene receptors from plants^23^.

A phylogenetic analysis of the ethylene receptors using the Arabidopsis (*Arabidopsis thaliana*) and tomato (*Solanum lycopersicum*) sequences shows that SlETR7 belongs to Sub-family II (Fig. 2b). Sub-family I members have three transmembrane regions in the ethylene binding domain, whereas, sub-family II members have an additional hydrophobic region ahead of the three helices that comprise the binding domain. Although, SlETR7 is predicted to lack this fourth transmembrane helix, it is similar to subfamily II receptors in that it has a longer stretch of amino acids ahead of the binding domain than the subfamily I receptors^24^. Thus, SlETR7 may be the first sub-family II member with only three transmembrane domains.

SlETR7 capacity to bind ethylene and function as an ethylene receptor that affects tomato development have never been tested. To test for the ability of SlETR7 protein to bind ethylene, the first 130 amino acids of SlETR7 fused to GST, called ETR7[1–130]GST (Supp. Fig. S3), were expressed in *P*. *pastoris*. This region of SlETR7 was chosen because it is predicted to contain the ethylene binding domain^25,26^. We then conducted ethylene binding assays using heterologous expression in yeast and radiolabeled ethylene. We also examined yeast expressing GST as a control (Supp. Fig. S3). As shown in Fig. 2c, the expression of ETR7[1–130]GST in yeast results in the formation of ethylene binding sites that are not seen with GST alone. Thus, SlETR7 directly binds ethylene.

### Alteration of *SlETR7* expression affects tomato seedling responses to ethylene

To further explore the in vivo roles of SlETR7 in tomato and determine if it functions as an ethylene receptor, we performed tomato transformation and obtained two KO SlETR7 lines, *KO-L1* and *KO-L2*, in which 5 and 11 bp were deleted in the site of sgRNA1, respectively (Supplementary Fig. S1B, C) and two over-expressing SlETR7 lines, *OE-L1 and OE-L2*, which showed a 20-fold and 50-fold increase in expression of *SlETR7* in leaves, respectively, compared to WT leaves (Supplementary Fig. S1D).

**Fig. 3:**
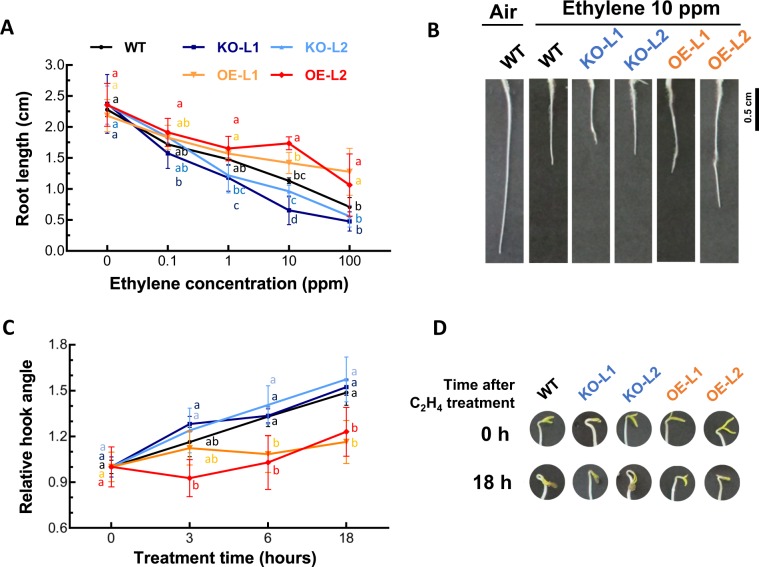
Effects of SlETR7 mutants on responses to ethylene in etiolated seedlings. **a**, **b** Changes in seedling root length of WT, *KO-L1, KO-L2, OE-L1* and *OE-L2* lines as a function of ethylene concentration. Treatments were conducted at 25 °C in the dark for 6 days. Data represent the mean ± SD, *n* ≥ 5. Tukey’s HSD tests were performed (*P* < 0.05) to compare tomato lines at each ethylene concentration, and significant differences are highlighted by different letters. **c**, **d** Hook angle changes as a function of time after a 1 ppm ethylene treatment. Four-day old seedlings of each line were treated with 1 ppm ethylene. Data represent the mean ± SD, *n* ≥ 5, relative to time “0” for each line, value that set at “1”. Dunn’s tests were performed for multiple comparison with *P* < 0.05 between tomato lines at each time, and significant differences are highlighted by different letters

To determine the effects of ethylene on these mutants, we examined the effects of exogenous ethylene on etiolated seedlings (Fig. 3). As shown in Fig. 3a, ethylene inhibited the growth of the WT roots consistent with the results of others^27^. However, neither the OE or KO lines had significant alterations in root growth inhibition compared to WT. The main exceptions to this was that KO-L1 was more responsive at 1 and10 ppm, and that OE-L2 was less responsive to ethylene than WT at 10 and 100 ppm, with a *P* < 0.05.

We were also interested in how these mutations affected the timing of ethylene responses. For this, we looked at the apical hook angle at various times after application of 1 ppm ethylene (Fig. 3b). In WT seedlings, the application of ethylene increased the angle of the apical hook with a measurable response within 3 h as reported previously^28^. Because the data were not normally distributed, we used a Dunn’s test for multiple comparison with *P* *<* 0.05. The KO seedlings showed a similar response as WT at all time-points. By contrast, application of ethylene had a little or no effect on the apical hook angle of OE lines. which had a 10- to 50-fold increase in *SlETR7* expression (Supp. S1D), Indeed, the hook angle was significantly lower than WT at 6 and 18 h in both OE lines. These results show that the OE lines are less responsive to ethylene.

### Alteration of *SlETR7* expression affects plant growth and flower transition in tomato

**Fig. 4:**
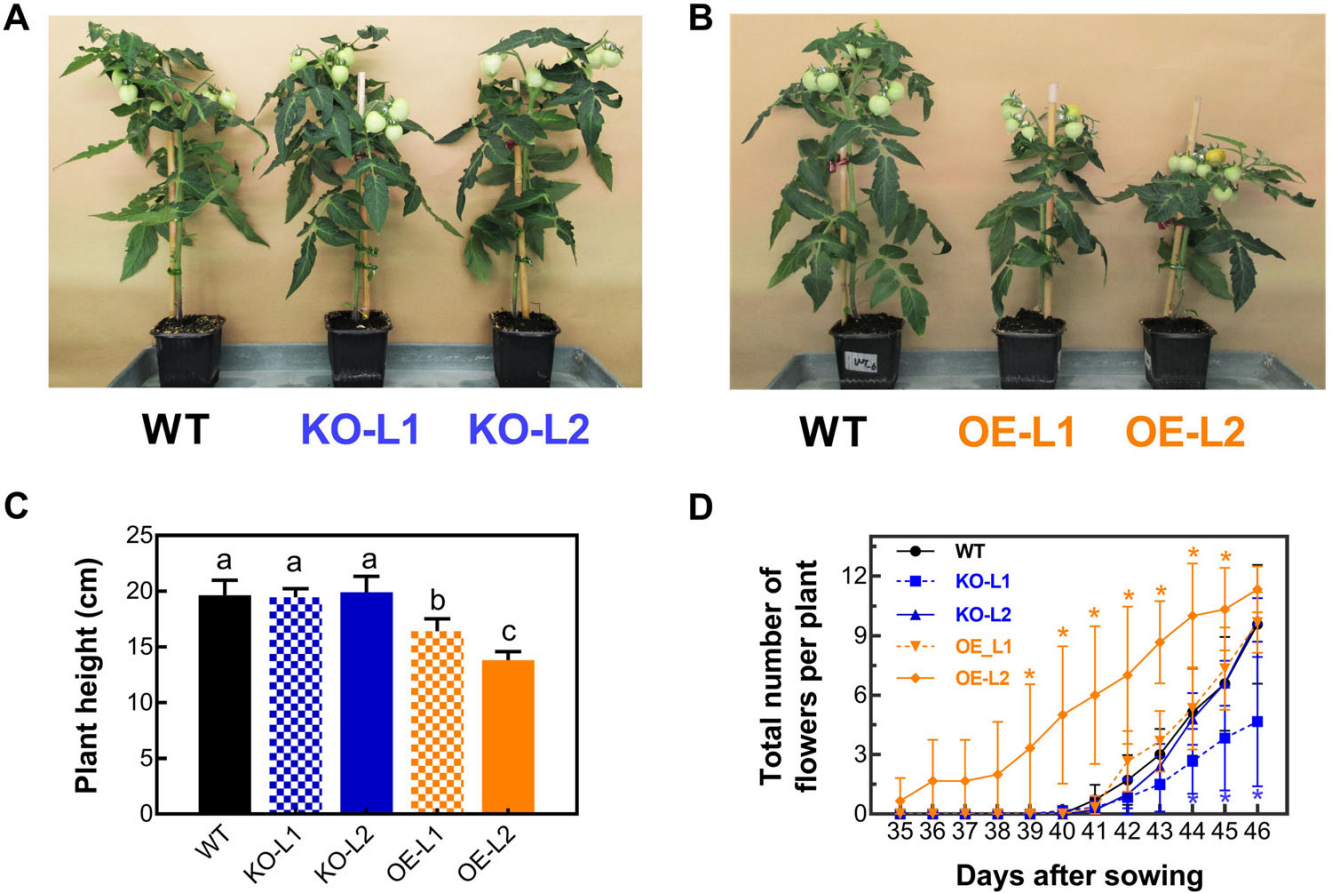
Effects of SlETR7 mutants on the plant growth and flower transition. **a**, **b** Representative WT, *KO-L1, KO-L2*, *OE-L1* and *OE-L2* lines after 80 days of cultivation. **c** Plant height of WT and *SlETR7* mutants. Data represent the mean ± SD, *n* = 8. The different letters in this graph mark significant differences at *P* *<* 0.05 using LSD multiple comparisons. **d** Number of flowers per plant as a function of the time after sowing. The flowers were counted from 35 days until 46 days after sowing. Data are the mean values ± SD, *n* = 8, “*” stands for a significant difference from WT at each time-point, using Tukey’s test (*P* *<* 0.05)

We wanted to know the effects of altering *SlETR7* levels on older plants. We first measured plant height of each line (Fig. 4a–c) and observed that the plant height of the two KO lines showed no significant difference (at *P* < 0.05 level) compared to WT (Fig. 4c). By contrast, OE-L1 was 16.5% shorter than WT and OE-L2 was 29.7% shorter than WT.

We also examined the timing of flowering transition in these plants. The KO-L1 line set flowers slower than WT (*P* *<* 0.05), whereas, the KO-L2 line was only slightly slower than WT. Conversely, OE-L2 start flowering 5 days before the WT, but OE-L1 was very similar to WT with only a slightly but significant (*P* *<* 0.05) more rapid onset of flowering compared to WT (Fig. 4d). The larger effect in OE-L2 is likely to be due to the higher expression of *SlETR7* in this line compare to OE-L1 (Supp. S1D). Together, these results indicate that SlETR7 affects growth and development.

### Changes in fruit size and ripening due to alteration of *SlETR7* expression

**Fig. 5:**
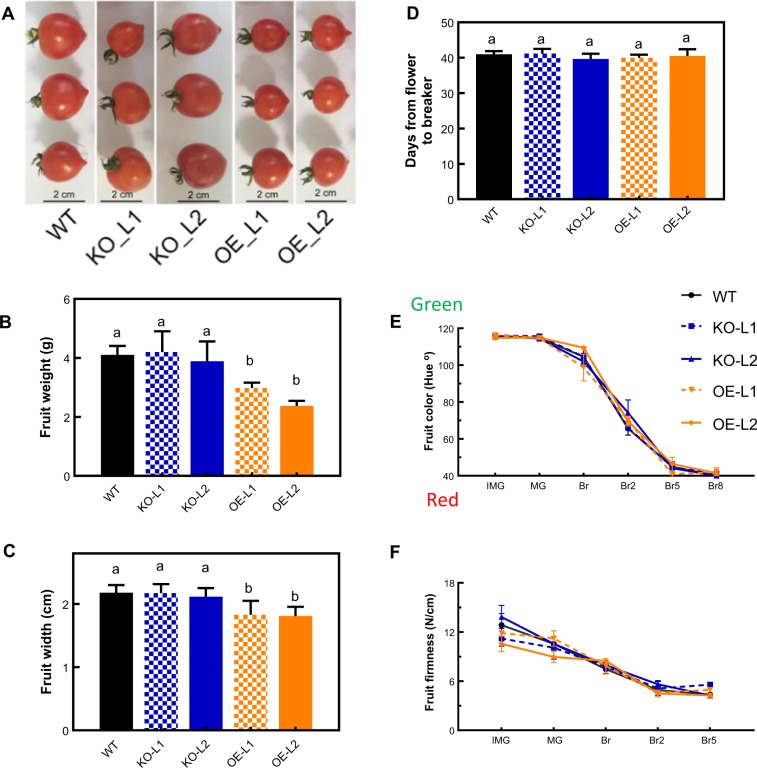
Effects of SlETR7 mutants on fruit size and ripening. **a**–**c** Differences of fruit size, weight and width of WT, *KO-L1, KO-L2, OE-L1* and *OE-L2* lines, 7-days after the breaker stage for each line. **d** Number of days between anthesis (flower) and fruit color change from green to yellow (breaker) stages; data represent the means ± SD, *n* = 8, different letter(s) marked significant differences using Tukey’s test (*P* *<* 0.05). **e** Changes in fruit firmness and **f** fruit color, as a function of the tomato lines. Data represent the mean ± SD, *n* = 8

We also observed that altering *SlETR7* expression altered fruit weight and width during fruit ripening (Fig. 5a–c). The changes in these phenotypes were visible only in the OE lines where there was a decrease in fruit weight and width compared to WT. By contrast, KO mutant fruits were not significantly different from WT fruits. For all lines, there was no change in fruit developing time from flower to fruit breaker stages (Fig. 5d). Similarly, no change was observed in typical tomato fruit ripening traits such as a decrease of firmness or the timing and extent of color change during ripening (Fig. 5e, f).

### Impact of SlETR7 mutants on ethylene synthesis and related gene expression

**Fig. 6:**
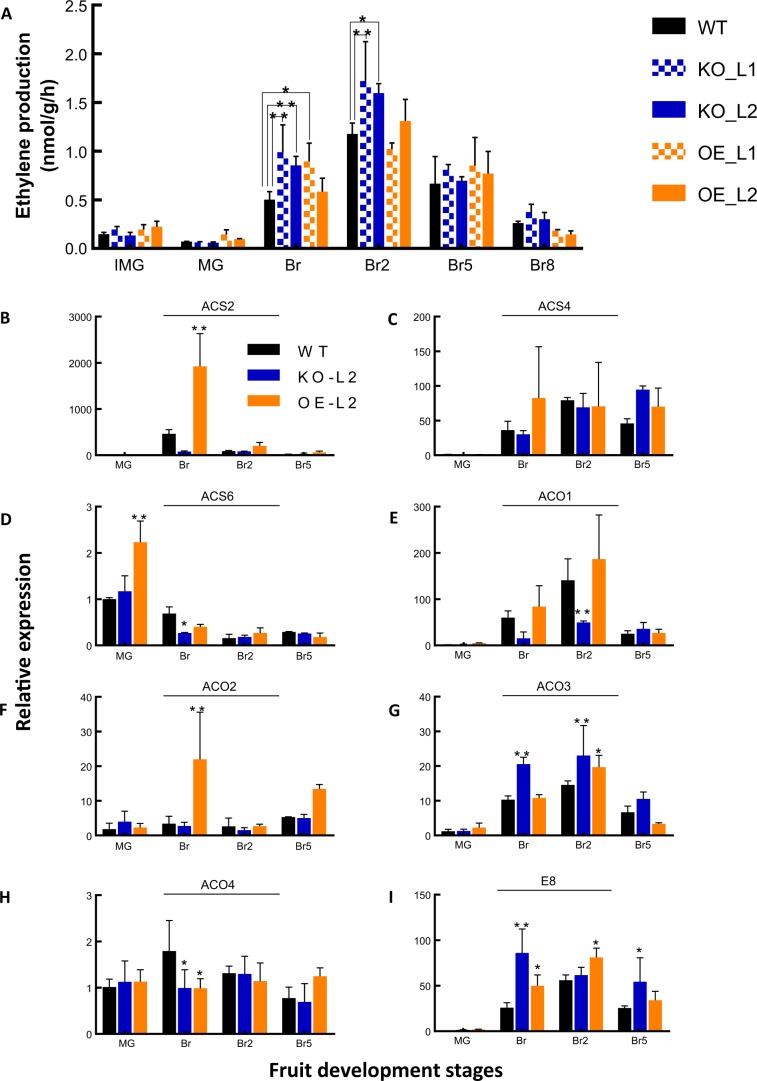
**a** Ethylene production at different fruit stages in tomato lines altered for the *SlETR7* expression. IMG stands for immature green, MG mature green, Br Breaker, Br2 to Br8 stand for Breaker + 2 days to Breaker + 8 days, respectively. Data are the mean ± SD, *n* = 3. Statistical differences were analyzed by Tukey’s tests in comparison to WT, at each development stage, “*” and “**” show *P* *<* 0.05 and *P* *<* 0.01, respectively. Other panels show the effects of *SlETR7* altered expression on gene expression related to ethylene synthesis. **b** *SlACS2*, an ACC synthase, **c** *SlACS4*, **d**, *SlACS6*, **e** *SlACO1*, an ACC oxidase, **f** *SlACO2*, **g** *SlACO3*, **h** *SlACO4*, and **i** the ethylene responsive gene *E8*, an ACO homolog. All data were obtained at four fruit development stages: MG, Br, Br2, and Br5. All data show mRNA levels relative to WT at MG stage. Data represent means ± SD, *n* = 3. Statistical analyses were performed using Tukey’s test comparing each line with WT, *P *<* 0.05, **P *<* 0.01

One of the traits of tomato fruit ripening is the burst in ethylene production around the breaker stage. We were therefore curious to know if SlETR7 affected ethylene production in ripening fruits. Unexpectedly, we observed that ethylene production at Br and Br2 stages in KO mutants was significantly higher than WT at the same stages (*P* *<* 0.05) (Fig. 6a). Generally, the OE mutants produced similar amounts of ethylene compared to WT except at Br where OE-L1 produced more ethylene than WT (Fig. 6a). This suggests that SlETR7 levels might affect feedback regulation of ethylene responsive genes, leading to changes in ethylene production.

Because of the changes observed in ethylene production, we checked if genes involved in ethylene production were altered in the KO or OE plants. For this we evaluated the KO-L2 and OE-L2 lines. ACC synthases (ACS2, ACS4, and ACS6) and ACC oxidases (ACO1 to ACO4) are known to be important for ethylene production over tomato ripening stages^15^. As shown in Fig. 6, there was an increase in the expression of *ACO3* (Fig. 6g) and an ACC oxidase homolog *E8* (*Solyc09g089580*) (Fig. 6i) in KO-L2 compared to WT. The OE-L2 plants also had a slightly increased level of these genes. This correlates with the higher ethylene production in the KO plants and somewhat higher levels of ethylene in the OE-L2 at Br (Fig. 6a). The expression of other ACOs and ACSs did not show this pattern. Other genes encoding ACSs and ACOs also showed alterations with several being upregulated in OE-L2 and downregulated in the KO-L2 plants. However, the patterns of change did not readily correlate with the alterations in ethylene production that were observed in Fig. 6a.

### Modulation of the expression of the seven *SlETRs* in SlETR7 mutants

**Fig. 7:**
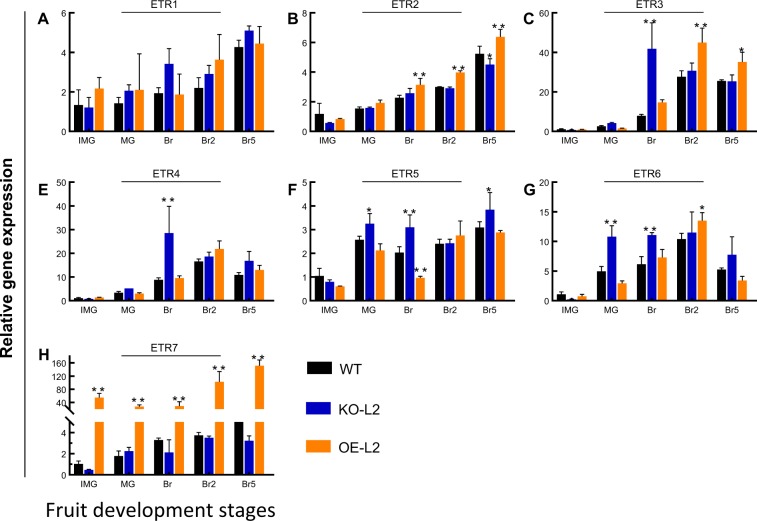
Effects of SlETR7 mutants on expression of the other SlETRs. **a**–**h** expression of *SlETR1, SlETR2, SlETR3, SlETR4, SlETR5, SlETR6*, and *SlETR7*, respectively at different stages of fruit development. IMG stands for immature green, MG mature green, Br Breaker, Br2 to Br8 stand for Breaker + 2 days to Breaker + 8 days, respectively. All data show mRNA levels relative to WT IMG stage for each studied gene. All the data represents mean ± SD, *n* = 3. Statistical analyses were performed using Tukey’s tests comparing each line to WT, *P < 0.05, **P < 0.01

It is known that down regulation of one *ETR* may be compensated by expression of other *ETRs* in both tomato^14^ and Arabidopsis^7^. Because this can obscure the role of an individual receptor and even mask the physiological effects of knocking out one receptor, we were interested to known whether there was similar compensation in the SlETR7 KO and OE plants. For this, we used qPCR to examine the transcript abundance of all seven receptor isoforms in WT, KO-L2, and OE-L2 lines (Fig. 7). Only *SlETR1* expression does not significantly change in either the KO or OE plants relative to WT (Fig. 7a). In KO-L2 plants, the levels of *SlETR3*, *SlETR4*, *SlETR5*, and *SlETR6* are upregulated in at least one stage of fruit ripening (Fig. 7c–g). This upregulation is likely to mask physiological changes due to the loss of SlETR7. In the OE-L2 line, *SlETR7* expression is 30–50-fold higher than WT (Fig. 7h) and this results in upregulation of *SlETR2*, *SlETR3*, and *SlETR6* during at least one stage of ripening (Fig. 7b, c, g) and downregulation of *SlETR5* at Br (Fig. 7f). The upregulation of several other *ETR*s may augment the effect of overexpressing *SlETR7*. Thus, there are complex changes in receptor transcript abundance when *SlETR7* is either knocked out or over expressed.

## Discussion

Ethylene receptors are key components for the initial steps of ethylene signal transduction in plant, and they show functional redundancy^12,14,24,29^. With the recent release of the tomato genome, a new potential ethylene receptor was discovered, SlETR7^15^ and its expression was shown to increase over the ripening period in existing RNAseq data sets^13^. The detailed analysis of expression of all seven ETRs, using higher time resolution than previous studies^11^, suggests that there are two groups of *SlETRs* that can be distinguished based on differences in their response to ethylene and in the timing of induction during ripening. It is interesting that, of the receptor genes that rise in the later stages of fruit ripening, *SlETR7* is the only one induced by exogenous ethylene. These differences in patterns of expression suggest that the two groups of receptors have different roles in controlling fruit ripening with the early induced genes having a larger role in the initiation of fruit ripening. While it is likely that the early induced genes are upregulated by increased levels of ethylene produced by the fruit, it is not yet known what signals are important for the increased levels of the other receptors that are upregulated later in fruit ripening. It would also be interesting to determine what traits are controlled by the ETRs induced later in ripening, including ETR7. Potential traits that could be regulated by one or more of these ETRs include fruit turgor pressure in late stages of ripening or during postharvest shelf life and seed maturation within the fruit.

No functional data was available for the newly discovered gene, *SlETR7*. Since SlETR7 has not been evaluated previously, we focused on this receptor to determine whether or not it is a functional ethylene receptor. Firstly, our detailed work on sequence discrepancies reveals an accurate *SlETR7* gene sequence. Based on results here, we predict SlETR7 belongs to the subfamily II, and that there are only three transmembrane domains at the N-terminus. If this is shown to be true experimentally, this would mean SlETR7 is the first subfamily II member to only have three transmembrane helices. Regardless of the answer to this, using heterologous expression of the N-terminal portion of SlETR7 showed that it directly binds to ethylene as shown previously for other SlETRs^7^ and this binding occurs to the N-terminal portion of the protein as predicted by bioinformatics.

Our results examining various plant traits confirm that SlETR7 is a functional receptor. Knocking out *SlETR7* induced small effects on seedlings responses to ethylene, but had no effect on plant height and fruit ripening. However, the KO lines showed increases in ethylene produced by the fruit, and in the levels of transcripts for genes that encode enzymes important for ethylene production. Thus, one distinct role of SlETR7 might be to repress the climacteric rise in ethylene production. This deserves further studies to decipher the feedback mechanism that involves SlETR7.

However, the limited changes in plant and fruit phenotypes caused by knocking out this receptor are likely due to functional compensation by the other ethylene receptors since several of the other isoforms had increased abundance upon removal of SlETR7. Such compensation has been observed before in various plant species^7,14^. By contrast, OE of *SlETR7* resulted in various physiological changes including reduced sensitivity to ethylene, shorter roots of etiolated seedlings, and smaller fruits. This further confirms that SlETR7 is a functional ethylene receptor. Similar observations have been observed upon OE of *OsETR2*^30^. It is likely that many of these alterations are due to decreased ethylene sensitivity. Additionally, the OE SlETR7 line with the strongest OE had a delay in flower transition while one of the KO SlETR7 lines showed a slight acceleration in flower transition. This regulation of flower transition by ethylene signaling has been described previously^31^ and the role of ethylene in flower development has been known for a long time^4^.

Kevany et al.^11^ reported earlier fruit ripening when the expression of either *SlETR4* or *SlETR6* was reduced. In the case of SlETR6, it is possible that antisense constructs, lacking specificity, may have affected ETRs other than the targeted ones; this will have to be evaluated further. By contrast, our results suggest that SlETR7 has only a minor role in fruit development and ripening with a few changes in fruit size, but no other obvious variation in fruit development and ripening speed. Indeed, there was neither a change in fruit softening nor in color turn between WT and KO SlETR7 lines. The functional compensation by the other receptors was further evidenced by the increased expression of several *SlETR*s upon removal of *SlETR7*. Such compensation is not limited to tomato since similar compensation has been reported in Arabidopsis^7^.

Globally, the observations reported here show that the *SlETR7* gene (Solyc05g055070) encodes a functional ethylene receptor. It remains to be determined how this receptor interacts with the other isoforms to control plant growth, development, and responses to stresses. Using studies on Arabidopsis as a model, it is likely that various combinatorial receptor null mutants will reveal interesting sub-functionalization of the tomato receptors involved in the regulation of important traits. Additional steps to further characterize SlETR7, would be to study the association with particular protein partners, beyond ETRs, at the level of the endoplasmic reticulum membrane; proteomics analyses could bring interesting information, as we now have powerful methods to detect low abundant proteins such as ETRs^32^.

## Supplementary information

Fig S1 Characterisation of ETR7 KO and OE lines

Fig S2 Nucleic acid and AA sequence of ETR7

Fig S3 Construct for ETR7 ethylene binding activity

Fig S4 Primers used in this study
