# Supplementary material for: Roles of SlETR7, a newly discovered ethylene receptor, in tomato plant and fruit development
Source: Hortic Res. 2020 Feb 1;7:17. doi: 10.1038/s41438-020-0239-y (PMC6994538; doi:10.1038/s41438-020-0239-y)
Supplement: Supplementary file 1 — Fig S1 Characterisation of ETR7 KO and OE lines [file 41438_2020_239_MOESM1_ESM.pptx]

## Slide 1
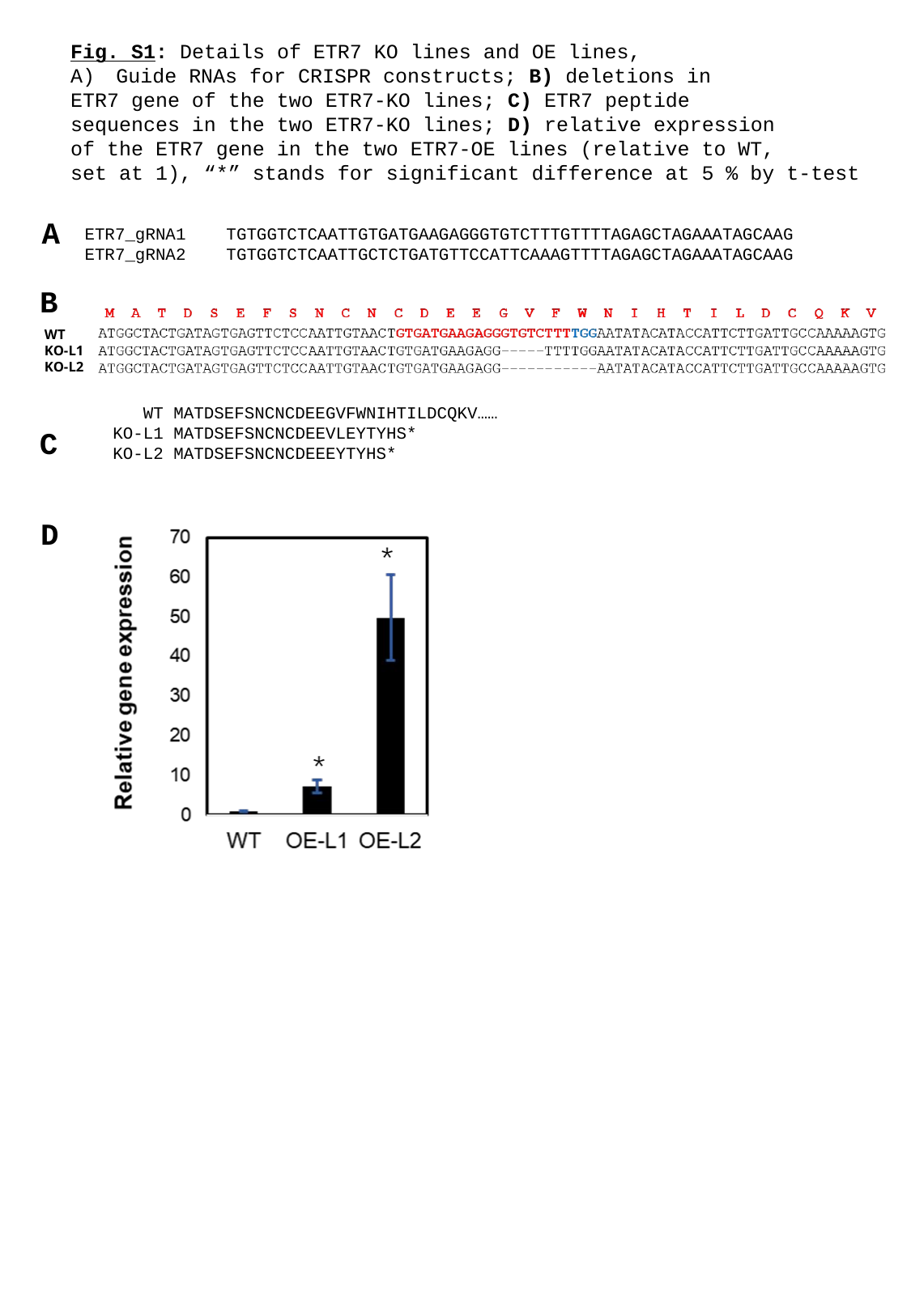

Fig. S1: Details of ETR7 KO lines and OE lines,
Guide RNAs for CRISPR constructs; B) deletions in
ETR7 gene of the two ETR7-KO lines; C) ETR7 peptide
sequences in the two ETR7-KO lines; D) relative expression
of the ETR7 gene in the two ETR7-OE lines (relative to WT,
set at 1), “*” stands for significant difference at 5 % by t-test
A
ETR7_gRNA1 TGTGGTCTCAATTGTGATGAAGAGGGTGTCTTTGTTTTAGAGCTAGAAATAGCAAG
ETR7_gRNA2 TGTGGTCTCAATTGCTCTGATGTTCCATTCAAAGTTTTAGAGCTAGAAATAGCAAG
B
WT
KO-L1
KO-L2
 WT MATDSEFSNCNCDEEGVFWNIHTILDCQKV……
 KO-L1 MATDSEFSNCNCDEEVLEYTYHS*
 KO-L2 MATDSEFSNCNCDEEEYTYHS*
C
D
