# Supplementary material for: Roles of SlETR7, a newly discovered ethylene receptor, in tomato plant and fruit development
Source: Hortic Res. 2020 Feb 1;7:17. doi: 10.1038/s41438-020-0239-y (PMC6994538; doi:10.1038/s41438-020-0239-y)
Supplement: Supplementary file 2 — Fig S2 Nucleic acid and AA sequence of ETR7 [file 41438_2020_239_MOESM2_ESM.docx]

**Supplementary Fig. S2:** Sequences of SlETR7; **A.** differences between the genome sequences; **B.** cloned SlETR7 sequence of nucleic acids; **C.** SlETR7 amino acid sequence

| Solyc05g055070.**2.4** | - | A | T | G | - | - | - | - | - | - | - | - | - | - | - | - | - | - | - | - | - | - | - | - | - | - | - | - | - | - | - | - | - | - | - | - | - | - | - | - | - | - | - | - | - | - | - | - | - | - | - | - | - | - | - | - | - | - | G | C | T | -6 |
| --- | --- | --- | --- | --- | --- | --- | --- | --- | --- | --- | --- | --- | --- | --- | --- | --- | --- | --- | --- | --- | --- | --- | --- | --- | --- | --- | --- | --- | --- | --- | --- | --- | --- | --- | --- | --- | --- | --- | --- | --- | --- | --- | --- | --- | --- | --- | --- | --- | --- | --- | --- | --- | --- | --- | --- | --- | --- | --- | --- | --- | --- | --- |
| Solyc05g055070.**3.3** | - | A | T | G | T | T | A | A | G | G | T | G | G | T | T | G | T | T | T | G | T | G | G | G | A | T | T | C | T | T | G | A | T | T | T | C | T | T | T | G | T | T | C | A | T | A | A | T | A | T | C | T | G | T | T | A | T | A | G | C | T | -60 |
| Solyc05g055070_mRNA_Micro-TOM | - | A | T | G | - | - | - | - | - | - | - | - | - | - | - | - | - | - | - | - | - | - | - | - | - | - | - | - | - | - | - | - | - | - | - | - | - | - | - | - | - | - | - | - | - | - | - | - | - | - | - | - | - | - | - | - | - | - | G | C | T | -6 |
|  |  |  |  |  |  |  |  |  |  |  |  |  |  |  |  |  |  |  |  |  |  |  |  |  |  |  |  |  |  |  |  |  |  |  |  |  |  |  |  |  |  |  |  |  |  |  |  |  |  |  |  |  |  |  |  |  |  |  |  |  |  |  |
| Solyc05g055070.**2.4** | - | A | C | T | G | A | T | A | G | T | G | A | G | T | T | C | T | C | C | A | A | T | T | G | T | A | A | C | T | G | T | G | A | T | G | A | A | G | A | G | G | G | T | G | T | C | T | T | T | T | G | G | A | A | T | A | T | A | C | A | T | -66 |
| Solyc05g055070.**3.3** | - | A | C | T | G | A | T | A | G | T | G | A | G | T | T | C | T | C | C | A | A | T | T | G | T | A | A | C | T | G | T | G | A | T | G | A | A | G | A | G | G | G | T | G | T | C | T | T | T | T | G | G | A | A | T | A | T | A | C | A | T | -120 |
| Solyc05g055070_mRNA_Micro-TOM | - | A | C | T | G | A | T | A | G | T | G | A | G | T | T | C | T | C | C | A | A | T | T | G | T | A | A | C | T | G | T | G | A | T | G | A | A | G | A | G | G | G | T | G | T | C | T | T | T | T | G | G | A | A | T | A | T | A | C | A | T | -66 |

**A**


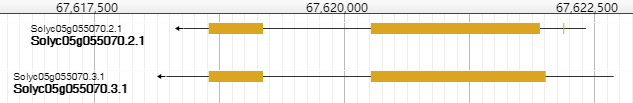


**B**

**>Sequence of ETR7 mRNA *(2238 bp)**

ATGGCTACTGATAGTGAGTTCTCCAATTGTAACTGTGATGAAGAGGGTGTCTTTTGGAATATACATACCATTCTTGATTGCCAAAAAGTGAGTGATTTCTTGATTGCAATTGCTTATTTTTCGATTCCACTCGAGTTGCTTTACTTTATTAGTTGCTCTGATGTTCCATTCAAATGGGTTCTTGTTCAATTCATTGCATTCATAGTTCTATGTGGATTGACTCATTTGCTCAATGGATTGACTTATAGTGCTCATCCTTCATTCCAATTGATAATGTCCTTAACCGTTGCGAAAATCCTAACCGCCCTTGTTTCTTGTGCAACTGCAATTACCCTTTTGACTTTGTTCCCTATGCTACTTAAAGTTAAGGTTAGAGAACTATTTTTGACTCAAAATGTGTTGGAGCTTGATCAAGAGGTTGGTATGATGAAGAAACAGAAAGAAGTGTATACTCATGTCCGAATGCTGACACGTGAGATTAGAAAGTCGCTTGATAAACATACTATATTGTATACTACTTTAGTTGAGCTTTCAAAGACATTGAATCTGCAGAATTGTGCTGTTTGGATGCCAAATGAGGATAGGTCATTGATGAACTTGACACACGGGTTAAGTCCCGGTTCTGCTGTAGAATACCATCGTTCACTTCCGATTGATGATCCGGATGTGTTAGAGATAACAAAGAACAAAGGAGTGAGAATTTTAAGACAAGATTCGGTTCTTGCAGCTGCAAGCAGTGGAGGGCCTGGTGAGCCATGTACTGTTGCAGCGATTAGGATGCCGTTGCTTTGTGCTTCGGATTTCAAAGGTGGGACACCTGAGTTGGTTGACACTCGATATGCTATTTTAGTTTTGGTTATTCCGGGTGCAAATGATGATTGTAGCCATAATGAGATGGAGATAGTGGAAGTAGTTGCTGATCAGGTGGCTGTGGCCCTATCCCACGCAACAGTTCTTGAAGAGTCACAATTAATGAGGGAGAAACTAGAAGCGAGGAATGGTTTGCTGCAACAGGCTAAGGAGAATGCTGTGAAGGCAAGCCAGGCAAGGAATTCGTTTCAGAAGGTAATGAACAATGGGATGAGACGGCCAATGCACTCGGTTTTGGGATTGCTTTCCATACTTCAAGATGAGAACACAAGCTCTAATCAGAAGATTATAATCGACACAATGGTGAGAACAAGCACCGTGCTGTCAAATTTAATAAACGATGCAATGGATATACCCGACAAAGACGAAGGGAGATTCCCAGTAGAAATGATGCCCTTTCAGCTGCATTCACTGATTAGAGAGGCTTCTTGTCTTGTTAAGTGCCTGTGTGTTTATAAGGGCTTTCGCTTTTCCACGGATGTTCCCAATTCTTTACCTAATCTGGTGATGGGTGATGAGAAGAGAACGTTTCAGGTTATACTTCATATGGTGGGACATCTATTGAATATCAGCTCCGGAAGGGGCTCCGTTGTATTCAAGGTTATTCTGGAGAGTGGAATCGAGGGCGGGAATGATAAGCTTCAGGGAGCAAGAAAACATAGCGTATTTGATGAATATGTTACCATAAAATTTGAGATTGAAGTTAGTCGTGGAGGTTCTCAAACAGATAGCTCAATCTCAACTTCTCACTTTGGCGGAAAGAGGTACAACAGCAAAGAGTTAAAGGAAGGCATGAGTTTCAGCATGTGCAAAAAGCTTGTTCAAATGATGCAGGGAAATGTATGGATGCCCTCAAATACCGATGGCCATGCACAAAAGATGACTCTTATTCTCCGATTTCTTAAACAGTCATCGTTCAGAAAACATATGTTTGAGCTTGTACATCCTTTGGAGCAAGCGATCTCAAGCTCAACGTTCAAAGGCCTCCAAGTTCTACTTGCTGATGATGACGACGTTAACAGAATGGTAACCAAAAAACTGCTTCAAAAACTAGGCTGCCAAGTGATTGCTGTTTCGTCTGGTTTTCAGTGCCTAAGTGCAATGGGACATTCAACAACTTCCATCCAAGTTGTCATTTTGGATCTTCACATGCCGGAAATGGACGGATTTGAAGTGACAACAAGGGTACGAAAATTCCACAGTCGTAGCTGGCCGTTGATCATAGCCTTATCTTCTACTTCAGAGCAACAAGTATGGGACAGATGTCTACAGGTTGGAATCAACGGTCTCATACGAAAGCCTGTTCTCCTGCAAGGAATGGCTGAAGAGCTTCAAAGAGTGTTACAAAGAGCTGGTGAAGGCTTTTAA

**C**

**>Sequence of ETR7 amino acid (746 AA)**

MATDSEFSNCNCDEEGVFWNIHTILDCQKVSDFLIAIAYFSIPLELLYFISCSDVPFKWVLVQFIAFIVLCGLTHLLNGLTYSAHPSFQLIMSLTVAKILTALVSCATAITLLTLFPMLLKVKVRELFLTQNVLELDQEVGMMKKQKEVYTHVRMLTREIRKSLDKHTILYTTLVELSKTLNLQNCAVWMPNEDRSLMNLTHGLSPGSAVEYHRSLPIDDPDVLEITKNKGVRILRQDSVLAAASSGGPGEPCTVAAIRMPLLCASDFKGGTPELVDTRYAILVLVIPGANDDCSHNEMEIVEVVADQVAVALSHATVLEESQLMREKLEARNGLLQQAKENAVKASQARNSFQKVMNNGMRRPMHSVLGLLSILQDENTSSNQKIIIDTMVRTSTVLSNLINDAMDIPDKDEGRFPVEMMPFQLHSLIREASCLVKCLCVYKGFRFSTDVPNSLPNLVMGDEKRTFQVILHMVGHLLNISSGRGSVVFKVILESGIEGGNDKLQGARKHSVFDEYVTIKFEIEVSRGGSQTDSSISTSHFGGKRYNSKELKEGMSFSMCKKLVQMMQGNVWMPSNTDGHAQKMTLILRFLKQSSFRKHMFELVHPLEQAISSSTFKGLQVLLADDDDVNRMVTKKLLQKLGCQVIAVSSGFQCLSAMGHSTTSIQVVILDLHMPEMDGFEVTTRVRKFHSRSWPLIIALSSTSEQQVWDRCLQVGINGLIRKPVLLQGMAEELQRVLQRAGEGF-
