# Supplementary material for: Roles of SlETR7, a newly discovered ethylene receptor, in tomato plant and fruit development
Source: Hortic Res. 2020 Feb 1;7:17. doi: 10.1038/s41438-020-0239-y (PMC6994538; doi:10.1038/s41438-020-0239-y)
Supplement: Supplementary file 3 — Fig S3 Construct for ETR7 ethylene binding activity [file 41438_2020_239_MOESM3_ESM.pptx]

## Slide 1
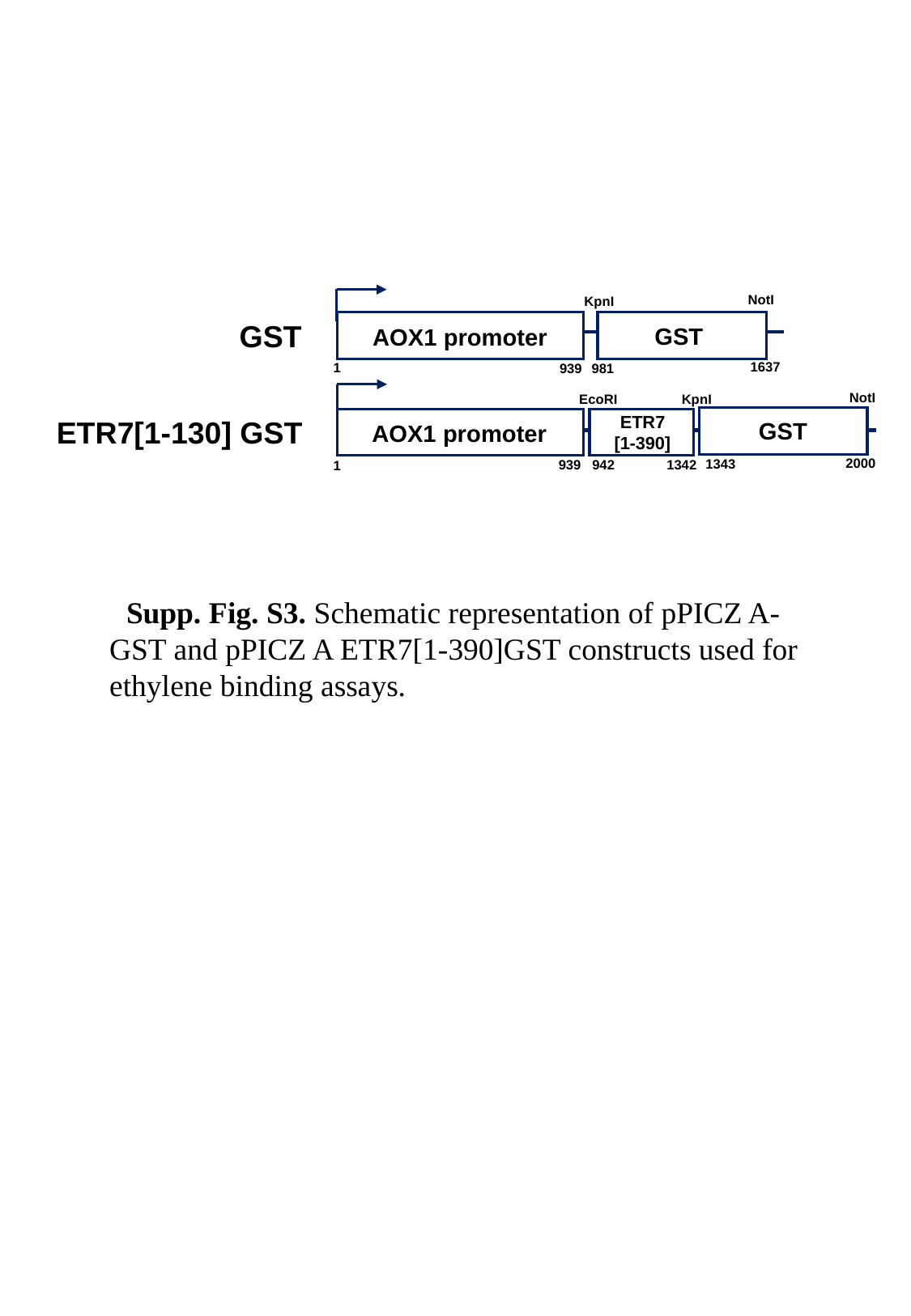

NotI
KpnI
GST
GST
AOX1 promoter
1637
1
981
939
NotI
KpnI
EcoRI
ETR7
[1-390]
ETR7[1-130] GST
GST
AOX1 promoter
2000
1343
939
1342
942
1
Supp. Fig. S3. Schematic representation of pPICZ A-GST and pPICZ A ETR7[1-390]GST constructs used for ethylene binding assays.
