# Supplementary material for: Roles of SlETR7, a newly discovered ethylene receptor, in tomato plant and fruit development
Source: Hortic Res. 2020 Feb 1;7:17. doi: 10.1038/s41438-020-0239-y (PMC6994538; doi:10.1038/s41438-020-0239-y)
Supplement: Supplementary file 4 — Fig S4 Primers used in this study [file 41438_2020_239_MOESM4_ESM.docx]

**Fig. S4** **qPCR primer list**

| Accession number | Primer name | Sequence (5’→3’) |
| --- | --- | --- |
| **Ethylene production** | | |
| Solyc07g049530 | SlACO1_F | GCCAAAGAGCCAAGATTTGA |
|  | SlACO1_R | TTTTTAATTGAATTGGGATCTAAGC |
| Solyc12g005940 | SlACO2_F | TTTATTACAAAGTGTGCGTCCCTA |
|  | SlACO2_R | CTCATTTTTGGGTATTAAAATATGTGT |
| Solyc07g049550 | SlACO3_F | TGATCAAATTGCAAGTGCTTAAA |
|  | SlACO3_R | ACCACACAACAATCACACACA |
| Solyc02g081190 | SlACO4_F | GGAGCCTAGGTTTGAAGCAA |
|  | SlACO4_R | AAACAAATTCCCCCTTGAAAA |
| Solyc01g095080 | SlACS2_F | TGTTAGCGTATGTATTGACAACTGG |
|  | SlACS2_R | TCATAACATAACTTCACTTTTGCATTC |
| Solyc05g050010 | SlACS4_F | CTCCTCAAATGGGGAGTACG |
|  | SlACS4_R | TTTTGTTTGCTCGCACTACG |
| Solyc08g008100 | SlACS6_F | CTCCTATGGTCCAAGCAAGG |
|  | SlACS6_R | CGACATGTCCATAATTGAACG |
| **Ethylene receptors** | | |
| Solyc12g011330 | SlETR1-F | GCCTTTTATCTTCCATCGTGGA |
|  | SlETR1-R | GATACTTCATTAGCAAGTCGTCAGCA |
| Solyc07g056580 | SlETR2-F | TGGCATTCCTGGTCGCTTA |
|  | SlETR2-R | TCTGCATGTGATTTGCAGGC |
| Solyc09g075440 | SlETR3-F | GCTTTGGCTCTGGATTTACCTATTC |
|  | SlETR3-R | TTCCCGCCACGTTTAAGAGA |
| Solyc06g053710 | SlETR4-F | GCCATACTGGTTTTGGTTCTACCTA |
|  | SlETR4-R | CCACAACCCTGACTATCTCAATTTC |
| Solyc11g006180 | SlETR5-F | TGTTCAGATGATGCAGGGAAAT |
|  | SlETR5-R | ATGAGTGTCATCCCCTGCG |
| Solyc09g089610 | SlETR6-F | AAAAGCCGGTGATCTCGGTA |
|  | SlETR6-R | AAACTAGAACAGGAAACGAAGTAGATGA |
| Solyc05g055070 | SlETR7-F | GGGTATGTTGGATCTTGAGGCA |
|  | SlETR7-R | GGCGGTTAGGATTTTCGCAAC |
| **Ethylene signal and related proteins** | | |
| Solyc09g089580 | SlE8_F | TGGCTCCGAATCCTCCCAGTCT |
|  | SlE8_R | GTCCGCCTCTGCCACTGAGC |
| **House keeping genes** |  |  |
| Solyc11g005330 | SlActin_F | TGTCCCTATTTACGAGGGTTATGC |
|  | SlActin_R | CAGTTAAATCACGACCAGCAAGAT |
| Solyc05g014470 | SlGAPDH_F | CTGCTCACTTGAAGGGTGGT |
|  | SlGAPDH_R | GACAATGTCCAGCTCTGGCT |
